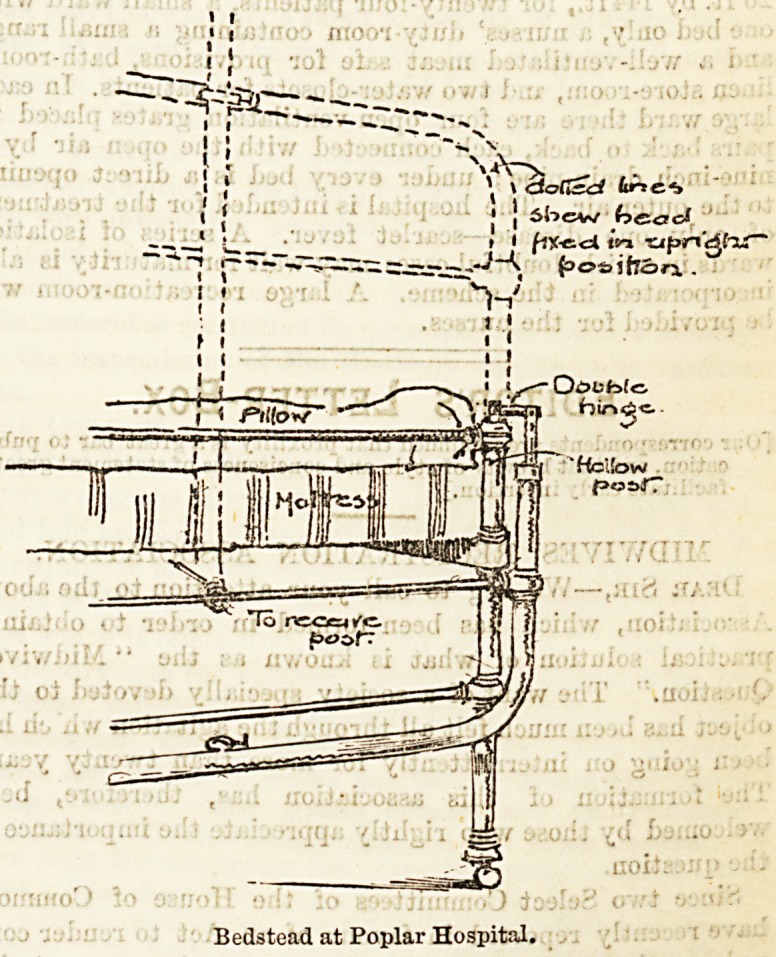# Appliances at Poplar

**Published:** 1894-01-20

**Authors:** 


### PRACTICAL DEPARTMENTS.

### APPLIANCES AT POPLAR (*continued*).

The varieties of bedsteads now in use in institutions and in private sick rooms are apparently endless, and new and ever-improving patterns are constantly coming to the fore. Many of the most modern inventions in this line are excellent in every respect, and well calculated to increase the comfort and convenience alike of patients and nurses.

Increased simplicity in form, and strong yet light construction, are the chief characteristics of the modern hospital bed. Everywhere now the delightfully comfortable and clean springs are taking the place of the lumpy flock beds which obtained in most institutions only a very short time since.

The beds at the Poplar Hospital are one and all provided with Lawson Tait springs, whilst a cleverly contrived arrangement of "fracture boards" is placed over these when necessary. Of these fracture boards we hope to give an illustration on a future occasion.

Bedstead at Poplar Hospital.

The present drawing gives a clear idea of a very excellent plan which has been adopted by the authorities at the Poplar Hospital to facilitate the administration of anæsthetics in the wards. It will be seen that the head of the iron bedstead is made with a double hinge, and can thus be allowed to fall entirely away, leaving the patient as conveniently placed as though on the operating table itself. The curved support for the patient over the head of the bed, of which the lower part is alone visible in the illustration, fits into a hollow post, and is therefore easily removed. The dotted lines in the accompanying sketch show the head fixed in its normal position.

Bedsteads made on a similar system are to be found at the Royal Eye Hospital, Southwark, where the head part is adapted for removal, and small operation tables can be fitted into the vacant posts.

The importance of good castors is now a generally-recognised fact, and we noticed that this point had been well considered in the Poplar bedsteads, and they could be moved with perfect ease, running lightly over the polished floors with little effort.

## Figures and Tables

**Figure f1:**